# Allergies: Ionizing Air Cleaners Zapped

**Published:** 2005-07

**Authors:** Ernie Hood

Ionizing air cleaners—those staples of infomercials and splashy magazine ads—are not only ineffective at removing contaminants from indoor air, but also may emit enough ozone to be a health concern. The effects may be even greater in people with respiratory problems, who make up 80% of the buyers of such devices. Those are the conclusions reached in tests of the units described in the May 2005 *Consumer Reports* (*CR*).

*CR* tested five units (including the top-selling Ionic Breeze from The Sharper Image) and confirmed results reported in October 2003 rating most of the air cleaners “poor” at removing dust and tobacco smoke from the indoor environment. This time around, pollen was added as well, with similarly disappointing results. The cleaners were also tested for generation of ozone, a respiratory irritant. The results showed that some of the least effective models also emitted potentially harmful ozone levels.

“We felt that it was particularly important to notify our subscribers that these air cleaners not only don’t remove particulates from the air, but they also put ozone into it,” says Jeff Asher, vice president and technical director of Consumers Union, the publisher of *CR*.

There is no regulatory standard for ozone emission by air cleaners; manufacturers claim to adhere to a voluntary standard of 50 parts per billion (ppb), a limit established by the Food and Drug Administration for medical devices. *CR* used Underwriters Laboratories Standard 867 to measure the units’ ozone levels from 2 inches away in a sealed polyethylene room. All five machines failed that test.

To more accurately reflect actual use conditions, *CR* also tested the devices in an open laboratory, from distances of 2 inches and 3 feet. Two units failed this this test; the other three (including the Ionic Breeze) produced levels of 26–48 ppb at 2 inches and 2–18 ppb at 3 feet—still high enough by *CR*’s estimation to be of concern. “The levels were not what I would call of great imminent risk,” says Asher, “but it was of significant risk in the sense of being in an indoor environment, where we just don’t need more ozone.”

The Sharper Image, which unsuccessfully sued *CR* over its 2003 report, has fired back, assailing the magazine’s credibility. In a 6 April 2005 press statement, CEO Richard Thalheimer called the article “irresponsible in the way it casually and unscientifically speculates about public health and safety. . . . We continue to emphatically disagree with Consumer Union’s methods in evaluating the Ionic Breeze.”

But health and engineering experts find the *CR* results troubling. “These levels make these devices inappropriate to use for asthmatic patients and for patients with respiratory disease,” says Peyton Eggleston, interim director of the Johns Hopkins Children’s Center.

Richard Shaughnessy, an environmental engineer at the University of Tulsa who has researched air cleaners for many years, concurs, pointing out that “not only are people with respiratory illnesses and asthma the population targeted by most of these air cleaners, they’re also the ones who are most likely to be adversely affected in terms of exposure to small amounts of ozone.”

## Figures and Tables

**Figure:**
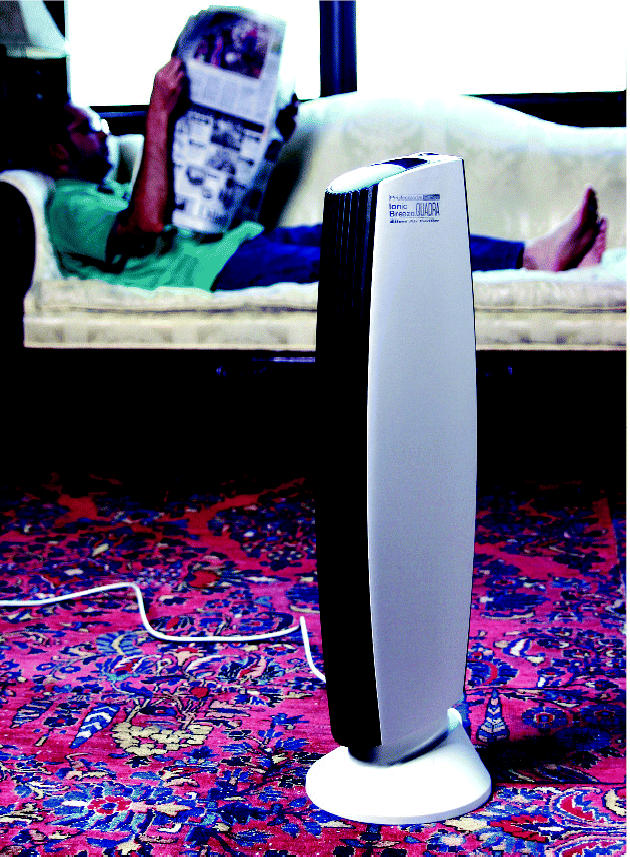
**Buyer beware.** Ionizing air cleaners don’t always live up to manufacturer claims and may emit harmful levels of ozone.

